# (*E*)-*N*′-(5-Bromo-2-hydroxy­benzyl­idene)-*p*-toluene­sulfonohydrazide

**DOI:** 10.1107/S1600536808037069

**Published:** 2008-11-13

**Authors:** Reza Kia, Hoong-Kun Fun, Hadi Kargar

**Affiliations:** a X-ray Crystallography Unit, School of Physics, Universiti Sains Malaysia, 11800 USM, Penang, Malaysia; b Department of Chemistry, School of Science, Payame Noor University (PNU), Ardakan, Yazd, Iran

## Abstract

The title compound, C_14_H_13_BrN_2_O_3_S, features an intra­molecular O—H⋯N hydrogen bond which generates an *S*(6) ring motif. The dihedral angle between the two benzene rings is 86.47 (6)°. Inter­molecular N—H⋯O and C—H⋯O inter­actions link neighbouring mol­ecules *via R*_2_^2^(13) ring motifs, forming one-dimensional extended chains along the *c* axis.

## Related literature

For background to sulfonamides, see: Kayser *et al.* (2004 ▶). For details of hydrogen-bond motifs, see: Bernstein *et al.* (1995 ▶). For related structures and applications see: Tabatabaee *et al.* (2007 ▶); Mehrabi *et al.* (2008 ▶); Ali *et al.* (2007 ▶); Tierney *et al.* (2006 ▶); Krygowski *et al.* (1998 ▶).
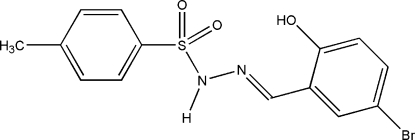


## Experimental

### 

#### Crystal data

C_14_H_13_BrN_2_O_3_S
*M* *_r_* = 369.23
Monoclinic, 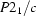

*a* = 15.8890 (3) Å
*b* = 9.8502 (2) Å
*c* = 9.8702 (2) Å
β = 105.475 (1)°
*V* = 1488.78 (5) Å^3^
*Z* = 4
Mo *K*α radiation
μ = 2.91 mm^−1^
*T* = 100.0 (1) K
0.45 × 0.34 × 0.31 mm

#### Data collection

Bruker SMART APEXII CCD area-detector diffractometer
Absorption correction: multi-scan (**SADABS**; Bruker, 2005 ▶) *T*_min_ = 0.308, *T*_max_ = 0.407
41486 measured reflections
7057 independent reflections
5961 reflections with *I* > 2σ(*I*)
*R*_int_ = 0.035

#### Refinement

*R*[*F* ^2^ > 2σ(*F* ^2^)] = 0.028
*wR*(*F* ^2^) = 0.079
*S* = 1.06
7057 reflections
199 parameters
H atoms treated by a mixture of independent and constrained refinement
Δρ_max_ = 1.28 e Å^−3^
Δρ_min_ = −0.59 e Å^−3^

### 

Data collection: *APEX2* (Bruker, 2005 ▶); cell refinement: *APEX2*; data reduction: *SAINT* (Bruker, 2005 ▶); program(s) used to solve structure: *SHELXTL* (Sheldrick, 2008 ▶); program(s) used to refine structure: *SHELXTL*; molecular graphics: *SHELXTL*; software used to prepare material for publication: *SHELXTL* and *PLATON* (Spek, 2003 ▶).

## Supplementary Material

Crystal structure: contains datablocks global, I. DOI: 10.1107/S1600536808037069/tk2325sup1.cif

Structure factors: contains datablocks I. DOI: 10.1107/S1600536808037069/tk2325Isup2.hkl

Additional supplementary materials:  crystallographic information; 3D view; checkCIF report

## Figures and Tables

**Table 1:** Hydrogen-bond geometry (Å, °)

*D*—H⋯*A*	*D*—H	H⋯*A*	*D*⋯*A*	*D*—H⋯*A*
N2—H1*N*2⋯O2^i^	0.851 (19)	2.002 (19)	2.8476 (13)	172.4 (19)
O1—H1*O*1⋯N1	0.84 (3)	1.94 (3)	2.6740 (14)	146 (3)
C7—H7*A*⋯O1^i^	0.93	2.60	3.3793 (15)	142
C10—H10*A*⋯Br1^ii^	0.93	2.91	3.8082 (12)	164
C12—H12*A*⋯O3^iii^	0.93	2.49	3.3691 (15)	158

Symmetry codes: (i) 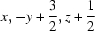; (ii) 
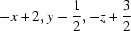
; (iii) 
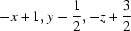
.

**Fig. 1.**
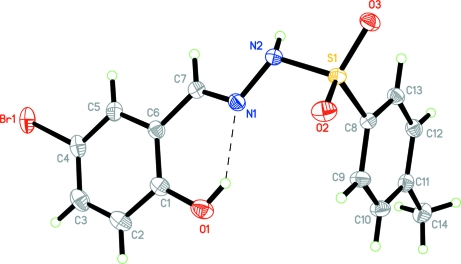
The molecular structure of (I), showing 50% displacement ellipsoids and the atomic numbering. The intramolecular hydrogen bond is shown as a dashed line.

**Fig. 2.**
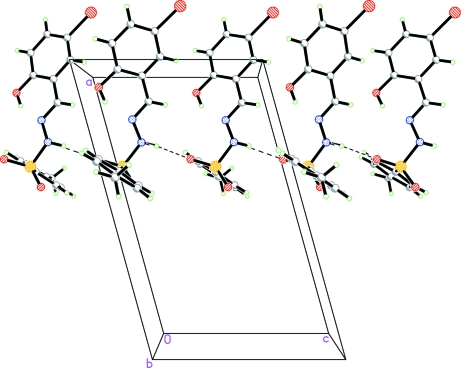
The crystal packing of (I) viewed down the b-axis, showing 1-D extended chains along the c-axis through N—H···O hydrogen bonding. Intermolecular hydrogen bonds are shown as dashed lines.

## supplementary crystallographic information

### Comment

Sulfonamides were the first class of antimicrobial agents to be discovered. They inhibit dihydropteroate synthetase in the bacterial folic acid pathway. Although their clinical role has diminished, they are still useful in certain situations, because of their efficacy and low cost (Krygowski *et al.*, 1998). Sulfonamides (e.g. sulfanilamide, sulfamethoxazole, sulfafurazole) are structural analogues of *p*-aminobenzoic acid (PABA) and compete with PABA to block its conversion to dihydrofolic acid. These agents are generally used in combination with other drugs to prevent or treat a number of bacterial and parasitic infections (Tierney *et al.*, 2006). With regard to all of the above, we report herein the crystal structure of the title compound, (I).

Bond lengths and angles in (I), Fig. 1, are comparable with those in related structures (Mehrabi *et al.*, 2008; Ali *et al.* 2007). An intramolecular O—H···N hydrogen bond is noted which generates a *S*(6) ring motif. The two benzene rings make the dihedral angle of 86.47 (6)°. Intermolecular N—H···O and C—H···O interactions link neighbouring molecules via *R*_2_^2^(13) ring motifs to form 1-D extended chains along the *c*-axis, Fig. 2 and Table 1. The crystal structure is further stabilized by intermolecular N—H···O, C—H···Br, C—H···O and π···π [*Cg1*···*Cg1* = 3.9548 (8) Å; symmetry code: 2 - *x*, 1 - *y*, 2 - *z*] interactions.

### Experimental

*p*-Tosylhydrazine (2 mmol) was added to a refluxing ethanolic solution (50 ml) of 5-bromosalicylaldehyde (2 mmol). The mixture was stirred for 2 h. After cooling, the colorless crystalline solid was isolated by filtration, washed with cold ethanol, and recrystallized from an ethanol solution of (I).

### Refinement

H atoms bound to O1 and N2 were located from a difference Fourier map and refined freely. The remaining H atoms were positioned geometrically and refined as riding model with C—H = 0.93 - 0.96 Å, and with *U*_iso_(H) = 1.2-1.5*U*_eq_(C). A rotating group model was used for the methyl group. The highest residual electron density peak (1.28 eÅ^-3^) was located 0.64 Å from Br1 and the deepest hole (-0.59 eÅ^-3^) was located 0.59 Å from Br1.

### Crystal data

**Table:**

C_14_H_13_BrN_2_O_3_S	*F*_000_ = 744
*M**_r_* = 369.23	*D*_x_ = 1.647 Mg m^−^^3^
Monoclinic, *P*2_1_/*c*	Mo *K*α radiation λ = 0.71073 Å
Hall symbol: -P 2ybc	Cell parameters from 9995 reflections
*a* = 15.8890 (3) Å	θ = 2.5–37.4º
*b* = 9.8502 (2) Å	µ = 2.91 mm^−^^1^
*c* = 9.8702 (2) Å	*T* = 100 (1) K
β = 105.475 (1)º	Block, colourless
*V* = 1488.78 (5) Å^3^	0.45 × 0.34 × 0.31 mm
*Z* = 4	

### Data collection

**Table:**

Bruker SMART APEXII CCD area-detector diffractometer	7057 independent reflections
Radiation source: fine-focus sealed tube	5961 reflections with *I* > 2σ(*I*)
Monochromator: graphite	*R*_int_ = 0.035
*T* = 100(1) K	θ_max_ = 36.0º
φ and ω scans	θ_min_ = 1.3º
Absorption correction: multi-scan(*SADABS*; Bruker, 2005)	*h* = −26→25
*T*_min_ = 0.308, *T*_max_ = 0.407	*k* = −16→16
41486 measured reflections	*l* = −16→16

### Refinement

**Table:**

Refinement on *F*^2^	Secondary atom site location: difference Fourier map
Least-squares matrix: full	Hydrogen site location: inferred from neighbouring sites
*R*[*F*^2^ > 2σ(*F*^2^)] = 0.028	H atoms treated by a mixture of independent and constrained refinement
*wR*(*F*^2^) = 0.079	*w* = 1/[σ^2^(*F*_o_^2^) + (0.0378*P*)^2^ + 0.5647*P*] where *P* = (*F*_o_^2^ + 2*F*_c_^2^)/3
*S* = 1.06	(Δ/σ)_max_ = 0.003
7057 reflections	Δρ_max_ = 1.28 e Å^−^^3^
199 parameters	Δρ_min_ = −0.59 e Å^−^^3^
Primary atom site location: structure-invariant direct methods	Extinction correction: none

### Special details

**Table:**

Experimental. The low-temperature data was collected with the Oxford Cyrosystem Cobra low-temperature attachment.
Geometry. All e.s.d.'s (except the e.s.d. in the dihedral angle between two l.s. planes) are estimated using the full covariance matrix. The cell e.s.d.'s are taken into account individually in the estimation of e.s.d.'s in distances, angles and torsion angles; correlations between e.s.d.'s in cell parameters are only used when they are defined by crystal symmetry. An approximate (isotropic) treatment of cell e.s.d.'s is used for estimating e.s.d.'s involving l.s. planes.
Refinement. Refinement of *F*^2^ against ALL reflections. The weighted *R*-factor *wR* and goodness of fit *S* are based on *F*^2^, conventional *R*-factors *R* are based on *F*, with *F* set to zero for negative *F*^2^. The threshold expression of *F*^2^ > σ(*F*^2^) is used only for calculating *R*-factors(gt) *etc*. and is not relevant to the choice of reflections for refinement. *R*-factors based on *F*^2^ are statistically about twice as large as those based on *F*, and *R*- factors based on ALL data will be even larger.

### Fractional atomic coordinates and isotropic or equivalent isotropic displacement parameters (Å^2^)

**Table:**

	*x*	*y*	*z*	*U*_iso_*/*U*_eq_	
Br1	1.191754 (8)	0.636650 (14)	1.149579 (15)	0.02941 (4)	
S1	0.645085 (17)	0.74186 (3)	0.60027 (3)	0.01571 (5)	
O1	0.91105 (6)	0.56509 (11)	0.60615 (10)	0.02610 (17)	
O2	0.67053 (6)	0.78504 (9)	0.47792 (9)	0.02295 (16)	
O3	0.57441 (5)	0.80755 (8)	0.63807 (9)	0.02088 (15)	
N1	0.80775 (6)	0.71072 (9)	0.72484 (10)	0.01769 (15)	
N2	0.73002 (6)	0.77064 (9)	0.73584 (10)	0.01728 (15)	
C1	0.97310 (8)	0.58383 (12)	0.72937 (13)	0.02124 (19)	
C2	1.05653 (9)	0.53277 (14)	0.74093 (15)	0.0272 (2)	
H2A	1.0683	0.4878	0.6652	0.033*	
C3	1.12225 (8)	0.54873 (14)	0.86523 (15)	0.0279 (2)	
H3A	1.1778	0.5144	0.8729	0.033*	
C4	1.10429 (8)	0.61625 (12)	0.97777 (14)	0.0237 (2)	
C5	1.02200 (8)	0.66843 (12)	0.96797 (13)	0.02211 (19)	
H5A	1.0110	0.7138	1.0441	0.027*	
C6	0.95532 (7)	0.65296 (11)	0.84359 (12)	0.01914 (18)	
C7	0.86985 (7)	0.70900 (11)	0.83885 (12)	0.01990 (18)	
H7A	0.8600	0.7445	0.9206	0.024*	
C8	0.62834 (7)	0.56588 (10)	0.59177 (11)	0.01615 (16)	
C9	0.66712 (8)	0.48649 (11)	0.50865 (12)	0.02087 (19)	
H9A	0.6983	0.5265	0.4518	0.025*	
C10	0.65842 (8)	0.34621 (11)	0.51211 (13)	0.0223 (2)	
H10A	0.6841	0.2924	0.4567	0.027*	
C11	0.61190 (7)	0.28427 (11)	0.59702 (12)	0.01949 (18)	
C12	0.57195 (8)	0.36678 (11)	0.67689 (13)	0.02032 (18)	
H12A	0.5393	0.3271	0.7318	0.024*	
C13	0.58016 (7)	0.50714 (11)	0.67562 (12)	0.01874 (17)	
H13A	0.5539	0.5612	0.7300	0.022*	
C14	0.60620 (9)	0.13240 (11)	0.60308 (15)	0.0257 (2)	
H14A	0.6230	0.0935	0.5250	0.039*	
H14B	0.5473	0.1063	0.5986	0.039*	
H14C	0.6446	0.1003	0.6894	0.039*	
H1N2	0.7171 (12)	0.7519 (19)	0.812 (2)	0.027 (4)*	
H1O1	0.8634 (18)	0.596 (3)	0.615 (3)	0.057 (7)*	

### Atomic displacement parameters (Å^2^)

**Table:**

	*U*^11^	*U*^22^	*U*^33^	*U*^12^	*U*^13^	*U*^23^
Br1	0.01916 (6)	0.02972 (7)	0.03454 (8)	−0.00490 (4)	−0.00119 (5)	0.00973 (5)
S1	0.01756 (10)	0.01561 (10)	0.01476 (10)	0.00098 (7)	0.00568 (8)	0.00033 (7)
O1	0.0248 (4)	0.0345 (5)	0.0198 (4)	0.0064 (3)	0.0074 (3)	0.0009 (3)
O2	0.0322 (4)	0.0216 (3)	0.0178 (4)	0.0002 (3)	0.0114 (3)	0.0031 (3)
O3	0.0191 (3)	0.0196 (3)	0.0246 (4)	0.0042 (3)	0.0069 (3)	−0.0005 (3)
N1	0.0164 (4)	0.0173 (3)	0.0206 (4)	0.0000 (3)	0.0071 (3)	0.0001 (3)
N2	0.0165 (4)	0.0191 (4)	0.0171 (4)	−0.0003 (3)	0.0059 (3)	−0.0025 (3)
C1	0.0217 (5)	0.0216 (4)	0.0217 (5)	0.0026 (4)	0.0081 (4)	0.0037 (4)
C2	0.0245 (5)	0.0295 (5)	0.0305 (6)	0.0072 (4)	0.0122 (5)	0.0035 (5)
C3	0.0208 (5)	0.0282 (5)	0.0359 (7)	0.0051 (4)	0.0095 (5)	0.0075 (5)
C4	0.0172 (4)	0.0233 (5)	0.0290 (6)	−0.0010 (4)	0.0035 (4)	0.0069 (4)
C5	0.0189 (5)	0.0213 (4)	0.0251 (5)	−0.0017 (4)	0.0042 (4)	0.0007 (4)
C6	0.0178 (4)	0.0181 (4)	0.0218 (5)	0.0002 (3)	0.0057 (4)	0.0017 (3)
C7	0.0181 (4)	0.0199 (4)	0.0219 (5)	−0.0005 (3)	0.0057 (4)	−0.0023 (4)
C8	0.0168 (4)	0.0161 (4)	0.0154 (4)	−0.0003 (3)	0.0040 (3)	−0.0008 (3)
C9	0.0253 (5)	0.0193 (4)	0.0211 (5)	−0.0006 (4)	0.0114 (4)	−0.0023 (4)
C10	0.0263 (5)	0.0184 (4)	0.0238 (5)	0.0005 (4)	0.0094 (4)	−0.0037 (4)
C11	0.0181 (4)	0.0174 (4)	0.0209 (5)	−0.0002 (3)	0.0015 (4)	0.0004 (3)
C12	0.0187 (4)	0.0201 (4)	0.0228 (5)	−0.0018 (3)	0.0067 (4)	0.0016 (4)
C13	0.0175 (4)	0.0197 (4)	0.0205 (4)	−0.0001 (3)	0.0075 (3)	−0.0002 (3)
C14	0.0291 (6)	0.0171 (4)	0.0286 (6)	−0.0001 (4)	0.0035 (5)	0.0009 (4)

### Geometric parameters (Å, °)

**Table:**

Br1—C4	1.8947 (13)	C5—H5A	0.9300
S1—O3	1.4292 (8)	C6—C7	1.4551 (16)
S1—O2	1.4363 (9)	C7—H7A	0.9300
S1—N2	1.6518 (10)	C8—C9	1.3907 (15)
S1—C8	1.7524 (10)	C8—C13	1.3943 (15)
O1—C1	1.3585 (16)	C9—C10	1.3900 (16)
O1—H1O1	0.84 (3)	C9—H9A	0.9300
N1—C7	1.2837 (15)	C10—C11	1.3971 (17)
N1—N2	1.3989 (13)	C10—H10A	0.9300
N2—H1N2	0.852 (19)	C11—C12	1.3972 (16)
C1—C2	1.3937 (17)	C11—C14	1.5008 (16)
C1—C6	1.4087 (16)	C12—C13	1.3891 (15)
C2—C3	1.392 (2)	C12—H12A	0.9300
C2—H2A	0.9300	C13—H13A	0.9300
C3—C4	1.388 (2)	C14—H14A	0.9600
C3—H3A	0.9300	C14—H14B	0.9600
C4—C5	1.3840 (17)	C14—H14C	0.9600
C5—C6	1.3995 (17)		
			
O3—S1—O2	120.16 (5)	C1—C6—C7	122.85 (11)
O3—S1—N2	103.88 (5)	N1—C7—C6	121.72 (10)
O2—S1—N2	106.13 (5)	N1—C7—H7A	119.1
O3—S1—C8	109.91 (5)	C6—C7—H7A	119.1
O2—S1—C8	109.02 (5)	C9—C8—C13	121.11 (10)
N2—S1—C8	106.82 (5)	C9—C8—S1	119.93 (8)
C1—O1—H1O1	108.2 (18)	C13—C8—S1	118.85 (8)
C7—N1—N2	115.19 (9)	C10—C9—C8	118.81 (10)
N1—N2—S1	114.34 (7)	C10—C9—H9A	120.6
N1—N2—H1N2	113.8 (13)	C8—C9—H9A	120.6
S1—N2—H1N2	110.0 (13)	C9—C10—C11	121.39 (10)
O1—C1—C2	118.13 (11)	C9—C10—H10A	119.3
O1—C1—C6	121.98 (10)	C11—C10—H10A	119.3
C2—C1—C6	119.89 (12)	C10—C11—C12	118.51 (10)
C3—C2—C1	120.33 (12)	C10—C11—C14	120.42 (11)
C3—C2—H2A	119.8	C12—C11—C14	121.07 (11)
C1—C2—H2A	119.8	C13—C12—C11	121.06 (10)
C4—C3—C2	119.57 (11)	C13—C12—H12A	119.5
C4—C3—H3A	120.2	C11—C12—H12A	119.5
C2—C3—H3A	120.2	C12—C13—C8	119.09 (10)
C5—C4—C3	120.94 (12)	C12—C13—H13A	120.5
C5—C4—Br1	118.48 (10)	C8—C13—H13A	120.5
C3—C4—Br1	120.58 (9)	C11—C14—H14A	109.5
C4—C5—C6	120.05 (12)	C11—C14—H14B	109.5
C4—C5—H5A	120.0	H14A—C14—H14B	109.5
C6—C5—H5A	120.0	C11—C14—H14C	109.5
C5—C6—C1	119.22 (11)	H14A—C14—H14C	109.5
C5—C6—C7	117.93 (10)	H14B—C14—H14C	109.5
			
C7—N1—N2—S1	−166.98 (8)	C5—C6—C7—N1	173.17 (11)
O3—S1—N2—N1	177.80 (7)	C1—C6—C7—N1	−7.24 (17)
O2—S1—N2—N1	−54.59 (9)	O3—S1—C8—C9	155.48 (9)
C8—S1—N2—N1	61.63 (8)	O2—S1—C8—C9	21.84 (11)
O1—C1—C2—C3	−179.66 (12)	N2—S1—C8—C9	−92.44 (10)
C6—C1—C2—C3	0.54 (19)	O3—S1—C8—C13	−28.24 (10)
C1—C2—C3—C4	−0.2 (2)	O2—S1—C8—C13	−161.87 (9)
C2—C3—C4—C5	−0.25 (19)	N2—S1—C8—C13	83.85 (9)
C2—C3—C4—Br1	178.81 (10)	C13—C8—C9—C10	−1.12 (18)
C3—C4—C5—C6	0.31 (18)	S1—C8—C9—C10	175.08 (9)
Br1—C4—C5—C6	−178.78 (9)	C8—C9—C10—C11	−0.07 (19)
C4—C5—C6—C1	0.06 (17)	C9—C10—C11—C12	1.54 (18)
C4—C5—C6—C7	179.67 (11)	C9—C10—C11—C14	−177.72 (12)
O1—C1—C6—C5	179.73 (11)	C10—C11—C12—C13	−1.87 (18)
C2—C1—C6—C5	−0.48 (17)	C14—C11—C12—C13	177.39 (11)
O1—C1—C6—C7	0.14 (17)	C11—C12—C13—C8	0.73 (18)
C2—C1—C6—C7	179.92 (11)	C9—C8—C13—C12	0.80 (17)
N2—N1—C7—C6	−178.58 (10)	S1—C8—C13—C12	−175.44 (9)

### Hydrogen-bond geometry (Å, °)

**Table:**

*D*—H···*A*	*D*—H	H···*A*	*D*···*A*	*D*—H···*A*
N2—H1N2···O2^i^	0.851 (19)	2.002 (19)	2.8476 (13)	172.4 (19)
O1—H1O1···N1	0.84 (3)	1.94 (3)	2.6740 (14)	146 (3)
C7—H7A···O1^i^	0.93	2.60	3.3793 (15)	142
C10—H10A···Br1^ii^	0.93	2.91	3.8082 (12)	164
C12—H12A···O3^iii^	0.93	2.49	3.3691 (15)	158

Symmetry codes: (i) *x*, −*y*+3/2, *z*+1/2; (ii) −*x*+2, *y*−1/2, −*z*+3/2; (iii) −*x*+1, *y*−1/2, −*z*+3/2.

## References

[bb1] Ali, H. M., Laila, M., Wan Jefrey, B. & Ng, S. W. (2007). *Acta Cryst.* E**63**, o1617–o1618.

[bb2] Bernstein, J., Davis, R. E., Shimoni, L. & Chang, N.-L. (1995). *Angew. Chem. Int. Ed. Engl.* **34**, 1555–1573.

[bb10] Bruker (2005). *APEX2* and *SAINT* Bruker AXS Inc., Madison, Wisconsin, USA.

[bb3] Kayser, F. H., Bienz, K. A., Eckert, J. & Zinkernagel, R. M. (2004). *Medical Microbiology*, pp. 1–20. Berlin: Thieme Medical.

[bb4] Krygowski, T. M., Pietka, E., Anulewicz, R., Cyranski, M. K. & Nowacki, J. (1998). *Tetrahedron*, **54**, 12289–12292.

[bb5] Mehrabi, H., Kia, R., Hassanzadeh, A., Ghobadi, S. & Khavasi, H. R. (2008). *Acta Cryst.* E**64**, o1845. 10.1107/S1600536808027219 PMC2960482 21201816

[bb6] Sheldrick, G. M. (2008). *Acta Cryst.* A**64**, 112–122. 10.1107/S0108767307043930 18156677

[bb7] Spek, A. L. (2003). *J. Appl. Cryst.* **36**, 7–13.

[bb8] Tabatabaee, M., Anari-Abbasnejad, M., Nozari, N., Sadegheian, S. & Ghasemzadeh, M. (2007). *Acta Cryst.* E**63**, o2099–o2100.

[bb9] Tierney, M., McPhee, S. Jr & Papadakis, M. A. (2006). *Current Medical Diagnosis & Treatment*, 45th ed., pp. 1–50. New York: McGraw–Hill Medical.

